# Thyroid Mediation of the Isoflavone Effects on Osteoporotic Bone: The Endocrine Interference With a Beneficial Outcome

**DOI:** 10.3389/fendo.2019.00688

**Published:** 2019-10-11

**Authors:** Branka Šošić-Jurjević, Vladimir Ajdžanović, Branko Filipović, Walter Severs, Verica Milošević

**Affiliations:** ^1^Department of Cytology, Institute for Biological Research “Siniša Stanković”, University of Belgrade, Belgrade, Serbia; ^2^College of Medicine, Pennsylvania State University, Hershey, PA, United States

**Keywords:** thyroid, bone, osteoporosis, isoflavones, genistein, daidzein

In this opinion article, we intend to elaborate on thyroid mediation of isoflavone effects mainly on the bone of aging males, which appears to have an important role but is still insufficiently analyzed in the existing literature. Namely, the consequences of exposure to endocrine-disrupting chemicals strongly depend on the timing of interaction and life stages of humans and animals, so we intended to highlight the thyroid-related mechanism underlying the beneficial effect of isoflavones on aged male bone.

Isoflavones are plant polyphenolic compounds dominantly present in legumes. Soybean products are the main source of isoflavones in the human diet, either consumed as food or as isolated soy protein, extracts, supplements, or purified compounds (1). In plants, isoflavones are mainly present as 7-O-β-d-glucosides (genistin and daidzin) and only in minor amounts in their free form as aglycones (genistein and daidzein) (1). The aglycones are biologically a more potent form, exerting hormone-like, antioxidant, anticarcinogenic, and osteoprotective actions (1). The potential use of isoflavones in the prevention and treatment of osteoporosis is even more actual considering that all current treatments manifest imperfections and bear the risks of side effects after prolonged usage (2).

Several epidemiological studies and clinical trials have revealed that supplementation with soy isoflavones improves bone health status in early and postmenopausal women (3). In parallel, aged men may have more bone-related benefits from isoflavone application than women, especially in the states of physiological or iatrogenic (prostate cancer therapy) androgen deficiency (4). Given that the number of men above the age of 70 is continuously increasing, the problem of male osteoporosis has been recognized as a major health-care challenge (5). Combined soy protein and isoflavone supplementation in men with type 2 diabetes mellitus led to a significant reduction in the bone resorption marker βCTX (6). However, epidemiological and clinical evidence regarding the bone-protective role of isoflavones in men is still scarce. In the meantime, research data on the mechanisms of action in well-defined models of male aging should provide useful insights into the therapeutic possibilities of isoflavones.

Thyroid hormones are known to play an important role in the regulation of bone metabolism, while isoflavones have been shown to interfere with thyroid homeostasis. Histological analysis of the effects of human hypothyroidism revealed low bone turnover with decreased osteoblast and osteoclast activities, resulting in increased mineralization without changes in bone volume (7). Crucially, the excess of thyroid hormones in hyperthyroidism results in stimulation of osteoclastogenesis and bone resorption (8). Gene expression of thyroid receptor (TR) α1 and β1 is confirmed in osteoclasts, but there are still doubts whether triiodothyronine (T3) stimulates osteoclast activity directly or whether these processes result from T3 actions in osteoblasts, osteocytes, and/or other bone cells (9, 10). When it comes to isoflavone effects in the context of thyroid status, *in vitro* and *in vivo* studies indicate different mechanisms of their action, whereas the changes of circulating thyroid hormone levels represent the outcome of all these effects. Namely, they inhibit thyroid peroxidase (TPO) activity by acting as competitive substrates for iodination (11). Genistein and daidzein inhibit binding of serum transport protein transthyretin (TTR) to thyroid hormones (12). However, the capacity of the thyroid system to compensate the consequences of isoflavone actions depends on numerous factors, such as insufficient iodine in the diet, co-exposure with other goitrogens, and age and sex (13–15). Human studies also indicate an association between increased risk of developing goiter on the one hand and high intake of soy-based foods combined with iodine deficiency on the other (11). The TPO inhibition was confirmed by identification of iodinated isoflavones in the urine of menopausal women upon isoflavone supplementation (16). However, although some clinical reports indicate the absence of the effect of isoflavones on thyroid hormone status (17), isoflavones tripled the risk of overt hypothyroidism development in postmenopausal women with subclinical hypothyroidism (18). There are still no available data regarding the potential remedial action of isoflavones on the bones in hyperthyroid states. It might be of importance for bone preservation, considering that both clinical and experimental studies coherently indicate that reduced bone density, osteoporosis, and increased fracture rate are associated with hyperthyroidism (8).

In an orchidectomized rat model of the andropause (15–16 months old Wistar males), subcutaneous application of various doses of genistein or daidzein (10 and 30 mg/kg b.m., daily for 3 weeks) suppressed thyroid activity, decreased thyroxin (T4) and T3, and elevated thyroid-stimulating hormone (TSH) in serum (19, 20). Such an effect of isoflavones was potentiated by both androgen deprivation and age (21, 22). At the same time, treatment of middle-aged rats with isoflavones (35 mg/kg b.m., daily for 4 weeks) elevated expression of T3-responsive genes, Dio1 enzyme activity, and concentration of T3 in the liver by interfering with TTR, which resulted in a mixed phenotype of systemic hypothyroidism and local (hepatic) tissue hyperthyroidism (12, 14). To note, thyroxin-binding globulin (TBG) is the major serum transport protein in humans but not TTR, while isoflavones do not compete with thyroid hormones for binding to TBG or albumin *in vitro* (12). However, TTR, in comparison with TBG, seems to be responsible for much of the immediate delivery of thyroid hormones to human tissues (23). Endogenous sex steroids differently influence the synthesis and stability of TBG in men and women (24). The treatment of middle-aged females with isoflavones under the same experimental setup remained without effects on thyroid homeostasis (13), which is in coherence with the fact that the females are less susceptible to perturbation of thyroid homeostasis (25).

In our middle-aged orchidectomized model, genistein and daidzein improved bone microarchitecture by increasing the cancellous bone formation, trabecular thickness, and trabecular number but decreasing the trabecular separation, all in the proximal tibial metaphysis (20, 26). Marked reductions in biochemical markers of bone formation, serum osteocalcin, and urinary Ca^2+^ concentrations were observed in comparison with those in orchidectomized controls (20, 26). In addition to this, isoflavones beneficially affected Ca^2+^/PI balance through modulation of parathyroid hormone/1.25(OH)2–vitamin D3/calcitonin balance (27). Most experimental studies also reported beneficial effects of soy isoflavones on the bone in male models (28, 29). Similarly, genistein (10 mg/kg b.m.) subcutaneously administered to ovariectomized females exerted a beneficial, bone-sparing effect by increasing bone mineral density and lowering serum osteocalcin concentration (30). Moreover, daidzein may be more efficient than genistein in the remediation of ovariectomy-induced bone loss (31).

Although the signs of systemic hypothyroidism coincided with higher bioavailability of thyroid hormones to bones in our model, TSH elevation was a favorable outcome within the frame of pituitary-thyroid mediation of bone-related isoflavone effects. TSH plays a direct role in the regulation of bone turnover independently of thyroid hormones (32). Heterozygous Tshr^+/−^ mice have normal levels of thyroid hormones and TSH but decreased bone density. Additionally, Tshr null mice have severe osteoporosis, and supplementation with T4 could not restore bone mass (32). Treatment of ovariectomized rats with low doses of TSH (insufficient to alter serum T3, T4, or TSH) also demonstrated that TSH independently prevented bone loss and increased bone mass (33). Human studies in postmenopausal women confirmed that subcutaneous injection of recombinant human TSH exerts both anti-resorptive and anabolic actions (34).

Besides general adrenocortical and thyroid functions interconnection in the regulation of energy balance, the interplay between glucocorticoids and hypothalamic-pituitary-thyroid axis may reflect adversely on morphofunctional characteristics of bone tissue. Stress conditions and glucocorticoids *per se* had been long ago established as the osteoporosis-promoting factors (35, 36). Moreover, glucocorticoids decrease thyrotropin-releasing hormone (TRH) gene expression in the hypothalamic paraventricular nucleus (PVN), as well as TSH serum concentrations in both rats and humans (37, 38). Given the previous elaboration of beneficial effects of high TSH on the bone (39), glucocorticoid indirect input (by lowering TSH) may also provoke the adverse effect on the bone homeostasis. Figure 1 provides a summary of data regarding thyroid mediation and other relevant endocrine aspects of isoflavone effects on osteoporotic bone in aged males. Genistein and daidzein were reported to be the strong inhibitors of 3β-HSD and cytochrome P450 21-hydroxylase enzymes, involved in the process of corticosteroid oogenesis (40). In our rat model, genistein and daidzein reduced both circulating levels of ACTH and corticosterone, thus reducing the risk of circulating glucocorticoid “pressure” on the osteoporotic bone (20, 26, 41). Thus, the isoflavone-glucocorticoid-TSH triad provides an additional favorable outcome in the context of pituitary-thyroid mediation of bone-sparing effects.

**Figure 1:**
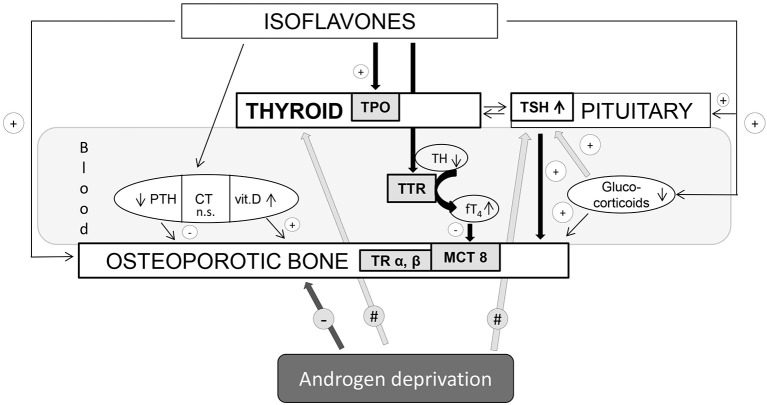
Thyroid mediation and other relevant endocrine aspects of isoflavone effects on osteoporotic bone in aged males. TPO, thyroid peroxidase; TH, thyroid hormone; TSH, thyroid-stimulating hormone; TTR, transthyretin; TR, thyroid receptor; MCT8, monocarboxylate transporter-8; PTH, parathormone; CT, calcitonin; vit. D, vitamin D. Arrows with “+” mean bone-protective effect; arrows with “–” mean bone-deteriorating effect; arrows with “#” mean local tissue changes upon androgen deprivation within the thyroid endocrine system.

Aside from the systemic effects of isoflavones, it is noteworthy to observe the possibility of their interference with bioavailability and signaling of thyroid hormones within the bone tissue. Genistein and daidzein may enhance T3-liganded, TR α and β-mediated transcription in a concentration-dependent manner (42). Moreover, although the role of thyroid hormone transporters in bone is still uncertain, the expression of monocarboxylate transporter-8 (MCT8) and l-amino acid transporter (LAT)-1 and LAT-2 was confirmed in osteoblasts and osteoclasts at all stages of cell differentiation (43). In parallel, it was suggested that genistein may bind to human MCT8 protein (44). Apart from thyroid hormone (TH) transporters, the relative activities of type 2 and 3 deiodinases (Dio2 and Dio3) determine intracellular availability of T3 in the bone, whereas Dio1 is not expressed in the skeleton (43). Genistein was shown to be a Dio1-specific inhibitor, without affecting the activity of Dio2 and Dio3 *in vitro* (45). The relevance of direct putative mechanisms of isoflavone action that might influence local tissue bioavailability and thyroid hormone signaling in bone still awaits further *in vivo* analyses.

In addition to the endocrine interference, the direct effect of isoflavones on the osteogenesis and osteoclastogenesis is also described, whereas selective estrogen receptor binding (46) represents one of the most suspected mechanisms. These compounds may also affect the paracrine activity in the bone, calcium channel signaling, and expression of osteoblastogenesis modulator core-binding factor 1 or receptor activator of nuclear factor-κB (RANK) expressed by osteoclasts, together with RANK ligand (RANKL) and osteoprotegerin (OPG) produced by osteoblasts (47).

The biopotency of aglycones is highly questioned at the moment because of their rapid metabolism, low serum concentration, and bioavailability upon supplementation (48). However, phase II of polyphenols conjugation does not always decrease their biological activity for a number of physiological endpoints (48). Certain isoflavone conjugates may represent an intracellular pool for generation and release of aglycones (49). Interestingly, the concentration of genistein in the thyroid and mammary gland tissue was reported to be markedly higher than in rat circulation (48). Apart from circulation, data regarding the concentration of isoflavones and their metabolites in bone tissue are still lacking, as well as the information regarding their potential bone-sparing effects, except for highly potent (*S*)-equol produced from daidzein by the gut microbiota. Great variations in response to pharmacotherapeutics, including isoflavones, point to a personalized approach. Definition of isoflavone metabolizing phenotypes as a strategy for identification of individuals might benefit from osteoporosis-preventive strategies using isoflavones.

## Conclusion

This opinion article is founded on the fact that soy isoflavones represent effective bioactive compounds that interfere with thyroid cascade and signaling at various points in a sex-and-age-dependent manner. A deeper understanding of the mechanisms involved in thyroid-mediated isoflavone action in the bone is even more important considering the complexity of regulation of bone remodeling, as well as the actuality of the bone disorders in advanced age. Potentially, therapeutic exploitation of biomedical essence presented here may inspire further research and development of isoflavone-based supplementation for men suffering from endocrine disorders and secondary osteoporosis. Basic and clinical research about isoflavone effects in thyrotoxicosis and associated bone pathology could be of particular interest.

## Author Contributions

All authors named have participated in the work in a substantive way. BŠ-J the Opinion concept creator, has written the majority of the article, analyzed the specific topics and interpreted the results of the research group in a broader context of literature. VA also contributed significantly to the writing of the article and provided the comprehensive approach to the scientific issue elaborated in the article. BF has included his focused expertise regarding bone microarchitecture and designed the figure. WS has carefully read and critically revised the manuscript for its scientific merit and intellectual content and has supplemented the literature survey. VM has supervised the work, carefully read the article for its scientific merit and intellectual content as well as supplemented the literature survey.

### Conflict of Interest

The authors declare that the research was conducted in the absence of any commercial or financial relationships that could be construed as a potential conflict of interest.
